# Overwintering Strategies and Post-Diapause Female Reproduction Fitness in the Willow Leaf Beetle *Plagiodera versicolora* (Coleoptera: Chrysomelidae)

**DOI:** 10.3390/insects16020140

**Published:** 2025-02-01

**Authors:** Jian Yan, Lin Zhang, Mingxuan Xu, Xiaofeng Zhang, Lvquan Zhao

**Affiliations:** 1 Bureau of Water Conservancy of Lianyungang, Lianyungang 222006, China; kailuwang@njfu.edu.cn; 2 Co-Innovation Center of Sustainable Forestry in Southern China, College of Forestry, Nanjing Forestry University, Nanjing 210037, China; zhanglin8220@njfu.edu.cn (L.Z.); xmx3210100088@njfu.edu.cn (M.X.); 3 Changsha Environmental Protection Vocational College, Changsha 410004, China; zxfrainbow@163.com

**Keywords:** *Plagiodera versicolora*, fecundity, post diapause, energy consumption

## Abstract

Diapause, a survival strategy utilized by many insects under severe environmental conditions, can generate costs that potentially affect post-diapause development and reproduction. The willow leaf beetle, *Plagiodera versicolora*, overwinters as an adult. This study investigated the cold hardiness-hardiness and energy utilization of female *P. versicolora*, and their impact on post-diapause reproductive fitness. The supercooling point exhibited seasonal temperature variation, with the lowest points occurring in January and February, coinciding with the relatively lower ambient temperatures. Lipid content demonstrated a pronounced decline at the onset of diapause (from November to December) and stabilized from December to March. Glycogen content also showed a sharp decrease from November to January, subsequently stabilizing at relatively constant levels. In addition, trehalose content increased significantly when temperatures dropped (from November to January) and then decreased as temperatures rose (from January to March). There were no significant differences in the time from pairing to successful mating for post-diapause females compared with non-diapause females. However, mating duration and the pre-oviposition period for post-diapause individuals relative to non-diapause individuals increased, coupled with a reduction in the oviposition period, total number of eggs, number of egg clutches, and number of eggs per clutch; however, most importantly, there was no notable change in egg-hatching success. These results suggest that the cold-hardiness strategy of *P. versicolora* falls within the freeze-avoidance category, with energy usage predominantly reliant on lipids and carbohydrates during diapause initiation. Our findings also highlight that, although post-diapause females are capable of nutrient replenishment, the energetic demands of diapause result in considerable negative impacts on post-diapause female reproductive fitness.

## Simple Summary

The willow leaf beetle *Plagiodera versicolora* is a serious pest of poplar and willow trees, on which it overwinters as an adult and then feeds post diapause. However, the cold-hardiness of the adults and their energy consumption during diapause, with resulting impacts on their post-diapause reproduction fitness, remain unclear. This study investigated the changes in supercooling point and levels of lipids, glycogen, and trehalose in diapause adults in response to seasonal temperature fluctuations, and compared the reproductive fitness of post-diapause females with non-diapause females. At the onset of diapause, the energy consumption of *P. versicolora* depended on lipid and carbohydrate stores, but, as diapause progressed, it shifted toward glycogen or other energy sources. Although post-diapause females were able to replenish nutrients lost during diapause, there was still a significant decrease in egg production and the number of clutches compared with non-diapause females. These results suggest that energy consumption during diapause leads to reduced post-diapause female reproduction fitness. Such insights are important for understanding the overwintering strategy of *P. versicolora* and could have implications for their ecology and population dynamics under climate change scenarios.

## 1. Introduction

Diapause, a form of dormancy determined by genetics and mediated by endocrine mechanisms, occurs at particular developmental stages and is influenced by both environmental and genetic factors [1,2,3]. Through diapause, insects are able to endure adverse environmental conditions and synchronize their life cycle with seasonal variations [4,5]. However, diapause typically extends over several months or even longer periods, during which most insects are unable to feed and, thus, replenish their nutritional stores. Therefore, protracted diapause presents a significant challenge in terms of energy consumption, because insects must sustain themselves without access to food resources [6].

Insects adopt two strategies to mitigate the energetic costs of sustaining this stage: (i) energy storage before diapause, and (ii) metabolic depression during diapause [6,7]. The accumulation of nutrients during the pre-diapause stage is essential, because any deficiency will reduce the chances of completing diapause successfully, thereby lowering prospects for survival and reproduction [8]. Metabolic suppression during diapause is a common phenomenon among insects, but the degree of inhibition can vary greatly across species and diapause strategies, such as a 15% metabolic rate decrease in adult Monarch butterflies versus a 90% drop in diapausing flesh fly pupae [9,10,11]. Energy reserves amassed during the diapause preparatory period serve to not only maintain basal metabolism during diapause, but also fuel post-diapause development, metamorphosis, flight, and reproduction. Consequently, the level of energy expenditure during diapause determines both survival rates throughout the dormant phase and post-diapause individual reproductive fitness. For example, goldenrod gall flies, *Eurosta solidaginis* Fitch, exposed to warmer temperatures during diapause showed increased energy consumption, resulting in a lower overwintering survival rate and post-diapause adult fecundity [12].

Aside from the energy consumption challenges during diapause, diapausing insects can sustain physiological damage and mortality as a result of freezing winter weather. Insects generally use three strategies (chill susceptibility, freeze tolerance, and freeze avoidance) to ensure survivability under low-temperature conditions [13]. Chill-susceptible (or freeze-avoiding) insects lower their supercooling point under stress, whereas freeze-tolerant types allow ice formation in extracellular spaces to survive below their supercooling points. Therefore, the supercooling point is considered the lower temperature limit of survival for insects in winter, serving as a key metric for assessing insect cold-hardiness [14,15]. When subjected to low growth temperatures during winter, insects synthesize and accumulate various low-molecular-weight compounds, such as polyols, sugars, and some amino acids, to enhance their cold tolerance during diapause [16]. For instance, in *Eurytoma plotnikovi*, changes in body sugars, including trehalose, myo-inositol, and sorbitol concentrations, occur seasonally, leading to variations in the supercooling point and significantly enhancing cold-hardiness [17].

The willow leaf beetle *Plagiodera versicolora* Laicharting (Coleoptera: Chrysomelidae) is widely distributed in Asia, Europe, and North Africa. Larvae and adults of *P. versicolora* feed on poplar and willow leaves and twigs, leaving only the midrib and a network of veins. Adult *P. versicolor* typically start to overwinter around late October or early November, and terminate overwintering in late March or late April the following year. To ensure safe overwintering, diapause females store more lipid and protein in their fat bodies during the diapause preparation phase compared with pre-reproductive females (unpublished data). Diapause and cold-hardiness are essential components for insect overwintering [1]. However, the patterns of cold tolerance and energy consumption in this beetle during diapause remain unclear. Additionally, although *P. versicolor* adults can replenish nutrition post diapause, whether energy usage during diapause affects the reproductive fitness of post-diapause females remains uncertain.

To clarify the cold-hardiness and energy consumption of *P. versicolora* during overwintering, and their impact on post-diapause reproduction fitness, we hypothesized that cold-hardiness for overwintering adults would vary with the season and the energy reserves would decrease as diapause progresses. We expected that the supercooling point fluctuates seasonally, with corresponding changes in trehalose content. Due to post-diapause females being able to replenish nutrient stores, we predicted that the energy usage during diapause will not result in considerable negative impacts on post-diapause female reproductive fitness such as the number of eggs, number of egg clutches, and hatching rate. These results will provide a scientific basis for better understanding the overwintering and reproduction strategies of *P. versicolora* and provide a scientific basis for improving the prediction of the population dynamics of this species.

## 2. Materials and Methods

### 2.1. Insect Source and Rearing

We collected the first batch of *P. versicolora* larvae from their host plant *Salix babylonica* in April 2022 in the Qixia District, Nanjing, Jiangsu Province, China (32.11° N, 118.95° E) and transported them to the laboratory at the Nanjing Forestry University, where they were fed fresh *S. babylonica* leaves in groups within plastic containers (5 cm diam. × 4 cm high) and maintained at a constant temperature of 25 ± 1 °C with a 16:8 light/dark (L/D) photoperiod. The leaves were replaced once each day until the larvae pupated. The pupae were transferred to a new container for adult emergence. Newly emerged adults were reared in groups within plastic containers. The females laid egg clutches on willow leaves, and the egg clutches were collected and transferred together with the willow leaves to Petri dishes, with moist filter paper placed at the bottom. The newly hatched larvae were transferred to new containers and were reared for one generation as described above.

The second batch of *P. versicolora* larvae was collected in October 2022 from the same area as the first batch of larvae. These larvae were also brought back to the laboratory and reared under natural environmental conditions following the methods described above. The emerged adults were then utilized for subsequent experiments.

### 2.2. Treatment of Overwintering Adults

Adult *P. versicolor* overwinter in the cracks of clumps of soil or beneath dead leaves from late October or early November. To simulate the overwintering conditions used in their natural habitat, adults (about 550 individuals) emerging from the second batch of *P. versicolora* larvae collected in October 2022 were segregated by sex and placed individually within plastic boxes. Each box contained a lightweight substrate comprising a mixture of peat moss, coco coir, perlite, and vermiculite. Holes were drilled in the lid and four walls of the container to maintain ventilation. The top of the plastic box was covered with absorbent cotton and a layer of moist filter paper was placed on the cotton to maintain an appropriate humidity level. The emerged adults were provided with fresh *S. babylonica* leaves until they ceased feeding and burrowed into the soil, indicating that they had entered diapause. Next, the plastic boxes were placed inside a carton, which was covered with a black plastic bag for shade and protection from rain. The cartons were then all placed on a veranda outside the laboratory.

### 2.3. Ambient Temperature Recording

A temperature recorder (GM1366,Jumaoyuan Technology Co., Ltd., Shenzhen, China) was placed on the veranda where the overwintering *P. versicolora* were situated and the ambient temperature was logged every 5 min. This captured the thermal environment experienced by the overwintering beetles throughout their overwintering period from November 2022 to April 2023. The data collected included daily mean temperatures and the daily maximum and minimum temperatures.

### 2.4. Determination of Supercooling Point

*Plagiodera versicolor* typically start overwintering around late October or early November, and terminate overwintering in late March or late April the following year. Therefore, the supercooling points of overwintering adults (17–30 individuals for females and males, respectively) were measured on the 28th day of each month from November 2022 to March 2023. These were measured using a supercooling point determinator (SUN-II intelligent insect-SCP determinator, SUN Company, Jinan, China). The beetles were placed on thermocouple electrodes connected to an auto-temperature-recording device within a programmable refrigerator. The temperature inside the refrigerator was decreased from 15 °C at a cooling rate of 0.5 °C per min down to −25 °C. The instrument recorded the insect body temperature every 1.6 s.

### 2.5. Biochemical Parameters

#### 2.5.1. Glycogen and Trehalose Content

Overwintering females and males (n = 10 for both groups) were placed in 1.5 mL centrifuge tubes to which 50 μL of 10% trichloroacetic acid (TCA) along with an appropriate amount of quartz sand was added. After sufficient homogenization, 950 μL of 10% TCA was added. After sufficient shaking, the mixture was centrifuged at 5000 rpm/min for 5 min at 4 °C. The supernatant was transferred to another 2000 μL centrifuge tube and the precipitate was dissolved in 1000 μL of 10% TCA. The dissolved precipitate was centrifuged again and the supernatant was combined with the supernatant collected after the first centrifugation; the residual precipitate was used for glycogen content determination. A 500 μL sample was transferred from the 2 mL centrifuge tube into a 1500 μL tube containing 1000 μL ethanol. This solution was then refrigerated at 4 °C for 16 h. Afterward, the supernatant was removed and centrifuged at 10,000 rpm/min for 20 min at 4 °C. The upper supernatant was removed and transferred to a 10 mL stoppered graduated test tube containing 1 mL of 0.15 mol/L sulfuric acid. After shaking and mixing, the mixture was heated in a boiling water bath for 10 min. Once cooled under running water, 1 mL of 30% KOH solution was added, and the solution was shaken thoroughly and reheated in boiling water for another 10 min. After cooling under running water, the sample was used for trehalose concentration determination.

The glycogen and trehalose contents were quantified by using the anthrone method [18]. The lower precipitate obtained from the above steps was fully dissolved in 1 mL distilled water before proceeding. For both trehalose and glycogen solutions, 50 μL of the solution was mixed with 950 μL distilled water, and 4 mL of 0.1% anthrone solution (formed by dissolving 1 g anthrone in 1000 mL of 80% sulfuric acid) was added while the test tube was in an ice water bath; the test tube was then heated to 100 °C for 10 min. After cooling under running water, the absorbance was measured at 620 nm, and concentrations were determined by comparing the absorbance with a standard glucose solution with known carbohydrate concentrations.

#### 2.5.2. Lipid Content

Lipid was extracted according to the method described by Lorenz [19]. A 50 μL sample was mixed with 1 mL acetic acid and 1 mL concentrated phosphoric acid, and then placed in a boiling water bath for 10 min. After cooling in running water, the mixture was combined with 2 mL of vanillin phosphate solution. Once the mixture had cooled, lipid levels were determined against cholesterol standards at 530 nm. We expressed glycogen, trehalose and lipid contents in contents per unit of lean tissue mass (mg g^−1^).

### 2.6. Female Reproductive Fitness

Diapausing adults were obtained from the second batch of larvae, and non-diapause beetles were newly emerged adults from the first batch of larvae in the present treatment. Preliminary field investigations found that overwintering adults started to emerge from the ground around mid-March, with feeding activities observed by April, indicating the termination of diapause. Therefore, from 20 March, all overwintering adults were transferred from the outdoor boxes to incubators maintained at a constant temperature of 25 ± 1 °C and with a 16:8 L/D photoperiod to terminate diapause. Non-diapause beetles had a pre-oviposition period of 7 days [20]. Seven days after adult emergence for non-diapause individuals and after transferring overwintering adults to the incubators from outdoors, mating pairs were established according to the method of Zhao et al. [20]. To compare the effects of energy expenditure during diapause on mating performance and reproductive fitness, females and males were only allowed to mate once. Mating behaviors were observed, and the time from pairing to successful mating (the time from moving the females and males to the container to the male inserting his genitalia into the female genitalia) and the mating duration (the time from the male inserting his genitalia into the female genitalia to the retraction of his genitalia) were recorded. After the females and males mated successfully, the male was removed and the female was reared separately following the rearing method described above. Once the female began to lay eggs, the egg clutches were collected and transferred together with the willow leaves to Petri dishes with moist filter paper placed at the bottom. The pre-oviposition period, oviposition period (the time from first oviposition to last oviposition), number of eggs, number of eggs clutches, number of eggs per clutch, egg-laying frequency, and hatching rate were all recorded.

### 2.7. Data Analysis

The experimental data were analyzed in SPSS 22.0 (IBM, Armonk, NY, USA). The supercooling point and biochemical composition of overwintering adults were analyzed using analysis of variance (ANOVA) with post hoc Tukey’s multiple range test [21]. ANOVA was also used to compare the time from pairing to successful mating, mating duration, number of eggs, number of egg clutches, number of eggs per clutch, pre-oviposition period, oviposition period, egg-hatching rate, and mean egg production every day between the non-diapause and post-diapause adults [22].

## 3. Results

### 3.1. Ambient Temperature

**Figure 1:**
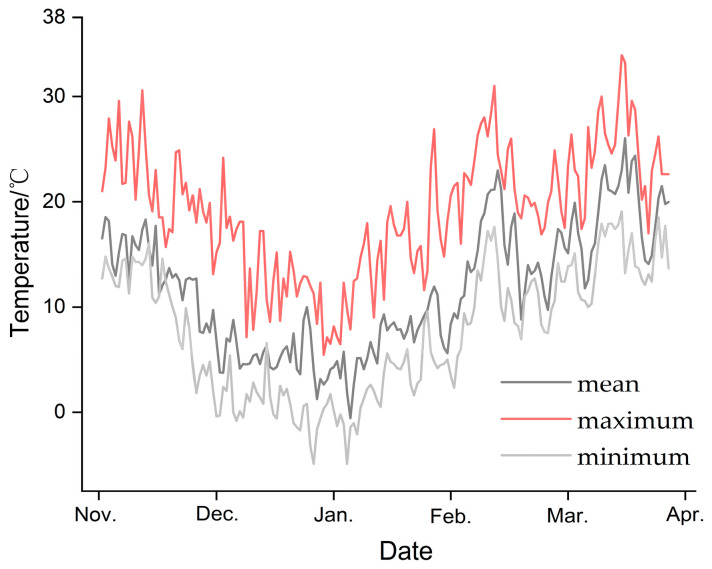
Ambient temperature of overwintering habitats of *Plagiodera versicolora* during the overwintering months from November 2022 to April 2023.

The mean ambient temperature in the Nanjing area of Jiangsu Province first decreased and then increased with the seasons (Figure 1). The mean ambient temperature in November was 14.9 °C, which decreased to 7.3 °C in December, 4.9 °C in January, and 2.2 °C in February, and then increased to 13.4 °C in March and 16.8 °C in April. The minimum temperature also showed a trend of first decreasing and then increasing, with the minimum temperature in January dropping to −3.2 °C.

### 3.2. Supercooling Point

**Figure 2:**
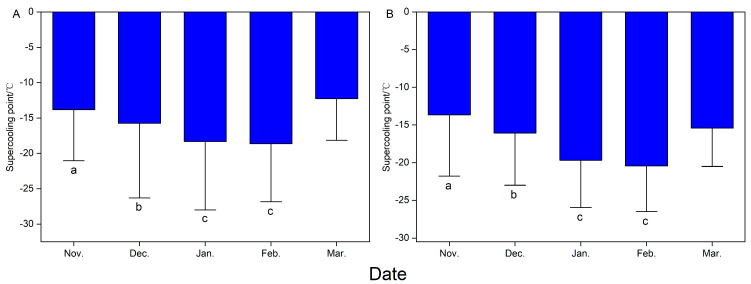
The supercooling point of overwintering adult *Plagiodera versicolora* during the overwintering months from November 2022 to March 2023. (**A**) Female; (**B**) male. Error bars represent the SE. Bars with different letters are significantly different from each other at *p* < 0.05 (n = 17–30).

The supercooling point of the overwintering adult *P. versicolora* showed significant changes with seasonal variation (female: F_4, 107_ = 7.42, *p* < 0.001; male: F_4, 106_ = 13.66, *p* < 0.001, Figure 2). The supercooling point of overwintering females first decreased and then increased with seasonal changes. The supercooling points in January and February were the lowest, and were significantly lower than those in November, December, and March (*p* < 0.05 in all cases, Figure 2A). A similar pattern was recorded in male adult *P. versicolora*, with the supercooling points in January and February again being the lowest, and significantly lower than those in November, December, and March (*p* < 0.05 in all cases, Figure 2B), whereas the supercooling point in March was similar to those in November and December (*p* = 0.72, *p* = 0.99, Figure 2B). 

### 3.3. Biochemical Parameters

**Figure 3:**
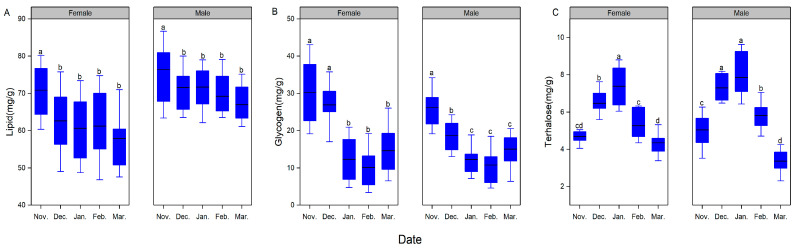
Seasonal patterns of biochemical parameters of overwintering adult *Plagiodera versicolora*. (**A**) lipid, (**B**) glycogen, and (**C**) trehalose. The top and bottom of each box represent the upper and lower quartiles, respectively; the horizontal line represents the mean; the vertical lines extend to the minimum and maximum values within 1.5 times the inter-quartile range. Different letters indicate significant differences from one another at *p* ˂ 0.05 (female: n = 10; male: n = 10, respectively).

Seasonal changes had a significant effect on the lipid content of adults (female: F_4, 49_ = 3.57, *p* = 0.013; male: F_4, 49_ = 3.52, *p* = 0.014, Figure 3A). As the seasons changed, the lipid content of female adults showed a gradually decreasing trend. The lipid content was highest at the beginning of overwintering in November, with 1.13, 1.16, 1.17, and 1.22 times more lipid compared with those in December, January, February, and March, respectively (*p* < 0.05 in all cases). However, there were no significant differences in the lipid content during December, January, February, and March (*p* > 0.05 in all cases). The lipid content of male adults showed a similar trend with seasonal changes.

Seasonal variation had a significant effect on the glycogen content of adults (female: F_4, 49_ = 21.23, *p* < 0.001; male: F_4, 49_ = 18.51, *p* < 0.001, Figure 3B). The amount of glycogen in females declined slightly from November to December, and then showed a sharp decrease from December to January, with only 0.41 and 0.46 times the glycogen content in January compared with that in November and December, respectively (*p* < 0.05). Subsequently, the glycogen content was maintained at a relatively stable level. For males, the glycogen content showed a significant decrease from November to January, with the glycogen content in December and January being only 0.65 and 0.43 times that in November, respectively (*p* < 0.05). From January to March, the glycogen content did not change significantly (*p* > 0.05).

Season also had a significant effect on the trehalose content of overwintering adults (female: F_4, 49_ = 31.14, *p* < 0.001; male: F_4, 49_ = 44.42, *p* < 0.001, Figure 3C). In females, the trehalose content showed a sharp increase from November to January, with 1.57 times and 1.86 times the amount of trehalose in December and January compared with that in November, respectively (*p* < 0.05), which differs from the decreasing trend in lipid and glycogen content during the same season. Subsequently, the trehalose content decreased significantly, and the trehalose content in February and March was only 0.71 and 0.6 times that in January (*p* < 0.05), respectively. The trehalose content of male adults showed a similar trend with seasonal changes.

### 3.4. Reproductive Fitness

**Figure 4:**
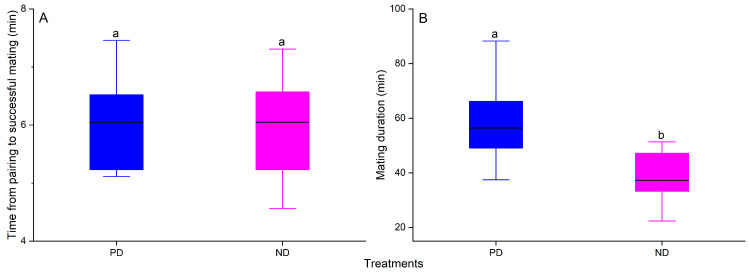
Comparison of the time from pairing to successful mating (**A**) and mating duration (**B**) of post-diapausing (PD, blue) and non-diapausing (NP, pink) adult *Plagiodera versicolora*. The top and bottom of each box represent the upper and lower quartiles, respectively; the horizontal line represents the mean; the vertical lines extend to the minimum and maximum values within 1.5 times the inter-quartile range. Different letters indicate significant differences from one another at *p* ˂ 0.05 (n = 10, respectively).

Overwintering had no significant effect on the time from pairing to successful mating but had a significant effect on mating duration (F_1, 19_ = 1.44, *p* = 0.25, Figure 4A; F_1, 19_ = 8.81, *p* < 0.0084, Figure 4B). The mating duration of post-diapause adults was 56.4 ± 17.9 min, which was significantly longer than that of non-diapause adults (37.2 ± 9.7 min, *p* < 0.05), with a 1.5 times longer mating duration in post-diapause adults compared with non-diapause adults.

**Figure 5:**
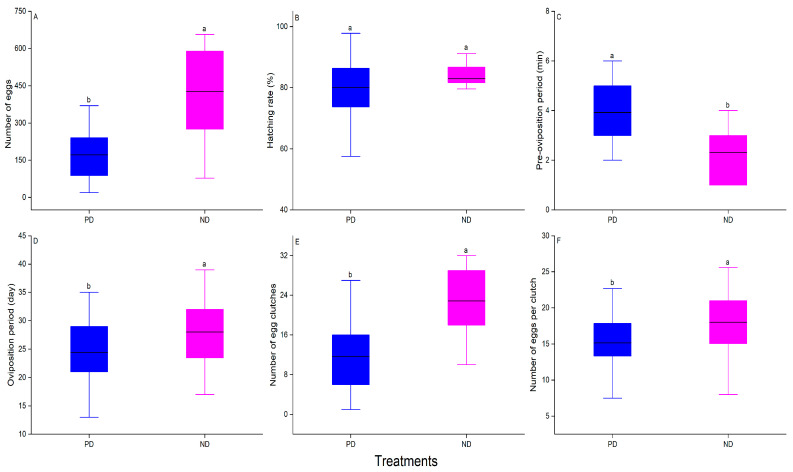
Comparison of the reproductive fitness of post-diapause (PD, blue) and non-diapause (NP, pink) adult female *Plagiodera versicolora*. (**A**) Number of eggs (n = 24–48); (**B**) hatching rate (n = 24–48); (**C**) pre-oviposition period (n = 24–49); (**D**) oviposition period (n = 24–49); (**E**) number of egg clutches (n = 23–48); and (**F**) number of eggs per clutch (n = 23–48). The top and bottom of each box represent the upper and lower quartiles, respectively; the horizontal line represents the mean; the vertical lines extend to the minimum and maximum values within 1.5 times the inter-quartile range. Different letters indicate significant differences from one another at *p* ˂ 0.05.

**Figure 6:**
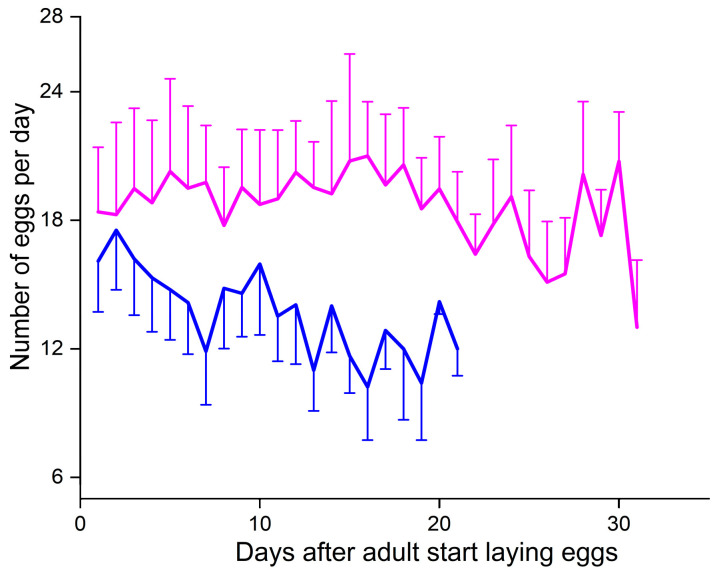
Mean (±SE) daily egg production of post-diapause (PD, blue) and non-diapause (NP, pink) adult female *Plagiodera versicolora*.

Overwintering also had a significant effect on fecundity, but did not significantly influence the egg-hatching rate (F_1, 70_ = 54.51, *p* < 0.001, Figure 5A; F_1, 70_ = 1.33, *p* = 0.25, Figure 5B). The fecundity of post-diapause females was 171.5 ± 14.4 (mean ± SE), which was 0.40 times of that of non-diapause females (426.2 ± 39.6) (Figure 5A). In addition, there were significant differences in the pre-oviposition period, oviposition period, number of egg clutches per female, and number of eggs per clutch between the two groups (F_1, 57_ = 526.98, *p* < 0.001, Figure 5C; F_1, 71_ = 12.9, *p* = 0.018, Figure 5D; F_1, 69_ = 32.02, *p* < 0.001, Figure 5E; F_1, 76_ = 46.65, *p* < 0.001, Figure 5F). The pre-oviposition period of post-diapause females was significantly longer than that of non-diapause females (Figure 5C). By contrast, the oviposition period, number of egg clutches per female, and number of eggs per clutch of non-diapause females were significantly greater than those of post-diapause females (*p* < 0.05 in all cases, Figure 5D–F). Importantly, the oviposition patterns of females in the two groups also differed (Figure 6). After the non-diapause females began to oviposit, the number of eggs oviposited remained relatively stable until ~20 days after the start of oviposition, when the number of eggs oviposited began to decrease, before then increasing and finally decreasing toward the end of oviposition. Unlike non-diapause females, the number of eggs produced by post-diapause females began to decrease slowly from when the females started laying eggs, and the number of eggs produced by post-diapause females was lower than that of non-diapause females throughout the oviposition period (*p* < 0.05 in all cases).

## 4. Discussion

Diapause is a widespread adaptive strategy that is crucial for the survival of overwintering insects and influences the distribution and abundance of many insect populations [5,23]. An insect in diapause will face two challenges, cold-hardiness and energy consumption, where an increase in energy consumption will reduce post-diapause individual fitness or even lead to insects being unable to safely overwinter, while cold or freezing temperatures will cause physiological damage and death [24,25]. Consistent with our expectations, the supercooling point showed a seasonal change trend similar to the changes in the seasonal temperature, with the lowest supercooling points occurring in January and February, coinciding with the lowest ambient temperature. Meanwhile, the lipid, glycogen, and trehalose contents also showed seasonal changes. The cold tolerance and levels of lipids, glycogen, and trehalose in *Hyphantria cunea* pupae during diapause also followed similar trends [21]. Contrary to our predictions, although *P. versicolora* post-diapause females are able to replenish nutrient stores, energy expenditure during diapause extended the mating duration and pre-oviposition period, reduced the number of egg clutches per female and number of eggs per clutch, and shortened the oviposition duration. This demonstrates that energy expenditure during diapause has a significant negative impact on the post-diapause reproduction fitness of female *P. versicolora*.

The main energy reserves for diapausing insects are lipids, glycogens, and proteins [7]. Our results showed that lipid content decreased significantly at the onset of diapause (from November to December), and then maintained a relatively stable level from December to March. Similarly, the glycogen content decreased significantly from November to January and then showed no significant change from January to March. These results suggested that energy expenditure in *P. versicolora* primarily relies on lipid and carbohydrate stores during early diapause, and then shifts to other energy sources as diapause progresses. Although diapause is a static state in insects, their metabolism is a dynamic process, and energy sources can be converted from lipids to glycogen or other energy reserves at specific stages [26,27]. Similar energy-utilization dynamics are observed in the diapausing pupae of *Sarcophaga crassipalpis*, where energy consumption is mainly dominated by lipids during the early stage of diapause, whereas energy demands shift toward glycogen or alternate substances as diapause progresses [28].

Based on physiological studies, it is generally believed that there are three main survival strategies for diapausing insects at low temperatures during winter: freeze-avoidant, chill-susceptible, and freeze-tolerant [29]. According to this classification, freezing-intolerant (or freezing-avoiding) insects are capable of lowering their supercooling point to improve their survival rate under cold-temperature stress. During the overwintering phase of *P. versicolora* adults, the trehalose content peaked during the coldest month (January), accompanied by a decrease in glycogen content. This indicates that overwintering adults of *P. versicolora* demonstrate temperature-dependent conversion between glycogen and trehalose correlated with seasonal changes in natural environmental conditions. For diapausing insects, glycogen can serve not only as an energy reserve, but also as a source for the synthesis of antifreeze and cryoprotectants to quickly respond to temperature changes, thereby improving the survival rate of insects during the overwintering period [30,31]. Variations in the trehalose content of overwintering *P. versicolora* adults correlated with seasonal temperature changes and also with changes in the supercooling point. Thus, the increased trehalose content in diapausing adults is a response to fluctuating environmental temperatures, likely acting as an antifreeze agent. However, the effect of changes in the supercooling points of overwintering adults on their cold tolerance was not investigated in the current study. Bemani et al. [32] reported that the increase in carbohydrates, such as trehalose, which accompanied the decrease in supercooling point, significantly improved the survival rate and cold-hardiness of diapause pupae of the pistachio fruit hull borer, *Arimania comroff*. Therefore, we speculate that *P. versicolora* overwintering adults lower their supercooling point by increasing their trehalose content, thereby improving their cold-hardiness during diapause and the overwintering period under natural environmental conditions; thus, they can be viewed as freeze-avoidant insects.

Generally, post-diapause adults will resume development and reproduction once diapause is terminated in spring. The mating behaviors of adults are influenced by various factors, such as age and mating experiences [20,22]. The present findings showed that the time from pairing to successful mating for non-diapause individuals was similar to that of post-diapause individuals, suggesting that the experimental procedure used ensured that the diapause termination and post-diapause development of diapause adults was achieved before the mating trials. Mating durations were notably longer for post-diapause females compared with their non-diapause counterparts. *P. versicolora* males and females do not achieve full sperm transfer with a single copulation [20], and the mating duration is correlated with female fertilization potential [33]. Therefore, for post-diapause *P. versicolora* adults, which have consumed a significant amount of energy during diapause, the extension of mating duration helps ensure the completion of female fertilization, thereby improving the reproductive success rate of the population. This is a crucial benefit that enables the population to quickly recover to pre-diapause levels.

While diapause enables insects to overcome harsh winter conditions, long-term diapause inevitably imposes energy consumption pressure, leading to poor longevity and reduced reproductive capacity of post-diapause individuals compared with non-diapause individuals [24]. For instance, the diapause of *Chilo partellus* adults resulted in a decrease in their body weight, which, in turn, reduced their post-diapause reproductive fitness [34]. The current results showed that diapause significantly prolonged the pre-oviposition period for post-diapause *P. versicolora* females, and diminished their reproductive output. Given the evident declines in lipid and glycogen stores during diapause, the delayed egg laying of post-diapause female enabled them to replenish their nutrient stores, thereby enhancing their potential reproductive performance. Previous results indicated that the reproductive fitness of *P. versicolora* is influenced by factors such as mating frequency and female age [20,22]. The current results showed that post-diapause females exhibit lower fertility compared with non-diapause females under identical mating conditions. Therefore, the above results might be related to the limited ovarian development of post-diapause females, caused by energy consumption during diapause. Despite the opportunity for nutritional replenishment, Tatar et al. [35] found in *Drosophila melanogaster* that diapause depletes essential nutrients, which cannot be replenished by adult feeding but are crucial for egg laying. Although a longer egg-laying period for non-diapause female *P. versicolora* helps to improve their fertility, post-diapause females not only laid fewer eggs, but also had a lower number of egg clutches per female and number of eggs per clutch compared with non-diapause females. More importantly, the mean daily egg production of post-diapause females was also lower than that of non-diapause females, indicating that, compared with non-diapause females, the ovarian development of post-diapause females is inhibited in terms of egg-laying ability within the same time period. In addition, the hatching rate of eggs of post-diapause females was similar to that of non-diapause females, indicating that diapause males were still able to successfully fertilize the females after a single mating. Therefore, we can exclude the possibility that differences in fertilization between post-diapause and non-diapause females were influenced by male fertilization differences. The fecundity or egg hatching of females was influenced by their mating age [36]. Our previous results indicated that delayed mating (21 days) of *P. versicolora* females did not significantly affect fecundity or egg hatching [22]. But the post-diapause females were about 150 days old in this study; therefore, we still could not exclude the possibility that the difference in reproduction fitness between the non-diapause and post-diapause females was influenced by the mating age of females. Further experiments are needed to verify the above speculation.

## 5. Conclusions

The cold-hardiness strategy of *P. versicolor* falls within the freeze-avoidance category, whereby the insects lower their supercooling point by increasing their trehalose content, thus improving their cold-hardiness during overwintering periods under natural environmental conditions. *P. versicolora* mainly relies on lipid and carbohydrate stores as energy sources during the early stages of diapause, whereas it changes to relying on carbohydrate or other energy stores during the middle or later stages of diapause. Importantly, although post-diapause females were able to replenish nutrients lost during diapause, energy consumption during diapause still has a significant negative impact on post-diapause female reproductive fitness. These results will provide a scientific basis for better understanding the overwintering and reproduction strategies of *P. versicolora*. Meanwhile, these results will provide essential information for determining the risk of damage to early-season trees based on the previous population size and for formulating pest management strategies for post-diapause populations.

## Data Availability

The original contributions presented in this study are included in the article. Further inquiries can be directed to the corresponding author.
